# Supplementary material for: HIVprotI: an integrated web based platform for prediction and design of HIV proteins inhibitors
Source: J Cheminform. 2018 Mar 9;10:12. doi: 10.1186/s13321-018-0266-y (PMC5845081; doi:10.1186/s13321-018-0266-y)
Supplement: Supplementary file 2 — Additional file 2. Source code of HIVProtI web server. [file 13321_2018_266_MOESM2_ESM.zip › HIVprotI_Source-code/hivprotI/weka-3-6-6/doc/weka/classifiers/trees/adtree/Splitter.html]

Splitter


---


|  |  |  |
| --- | --- | --- |
| **Overview** \| **Package** \| **Class** \| **Tree** \| **Deprecated** \| **Index** \| **Help** \| **Weka's home** | |  |
| **PREV CLASS**   **NEXT CLASS** | **FRAMES**    **NO FRAMES**     **All Classes** |  |
| SUMMARY: NESTED \| FIELD \| CONSTR \| METHOD | DETAIL: FIELD \| CONSTR \| METHOD |  |


---


## weka.classifiers.trees.adtree Class Splitter

```
java.lang.Object
  weka.classifiers.trees.adtree.Splitter
```

**All Implemented Interfaces:**: java.io.Serializable, java.lang.Cloneable, RevisionHandler

**Direct Known Subclasses:**: TwoWayNominalSplit, TwoWayNumericSplit

---

``` public abstract class Splitter extends java.lang.Object implements java.io.Serializable, java.lang.Cloneable, RevisionHandler ```

Abstract class representing a splitter node in an alternating tree.

**Version:**
:   $Revision: 1.5 $

**Author:**
:   Richard Kirkby (rkirkby@cs.waikato.ac.nz)

**See Also:**: Serialized Form

---

| **Field Summary** | |
| --- | --- |
| `int` | `orderAdded`             The number this node was in the order of nodes added to the tree |


**Constructor Summary**

`Splitter()`


| **Method Summary** | |
| --- | --- |
| `abstract  java.lang.String` | `attributeString(Instances dataset)`             Gets the string describing the attributes the split depends on. |
| `abstract  int` | `branchInstanceGoesDown(Instance i)`             Gets the index of the branch that an instance applies to. |
| `abstract  java.lang.Object` | `clone()`             Clones this node. |
| `abstract  java.lang.String` | `comparisonString(int branchNum, Instances dataset)`             Gets the string describing the comparision the split depends on for a particular branch. |
| `abstract  boolean` | `equalTo(Splitter compare)`             Tests whether two splitters are equivalent. |
| `abstract  PredictionNode` | `getChildForBranch(int branchNum)`             Gets the child for a branch of the split. |
| `abstract  int` | `getNumOfBranches()`             Gets the number of branches of the split. |
| `abstract  ReferenceInstances` | `instancesDownBranch(int branch, Instances sourceInstances)`             Gets the subset of instances that apply to a particluar branch of the split. |
| `abstract  void` | `setChildForBranch(int branchNum, PredictionNode childPredictor)`             Sets the child for a branch of the split. |

**Methods inherited from class java.lang.Object**

`equals, getClass, hashCode, notify, notifyAll, toString, wait, wait, wait`

**Methods inherited from interface weka.core.RevisionHandler**

`getRevision`

**Field Detail**

### orderAdded

```
public int orderAdded
```

:   The number this node was in the order of nodes added to the tree


**Constructor Detail**

### Splitter

```
public Splitter()
```


**Method Detail**

### getNumOfBranches

```
public abstract int getNumOfBranches()
```

:   Gets the number of branches of the split.

    :   **Returns:**: the number of branches

---


### branchInstanceGoesDown

```
public abstract int branchInstanceGoesDown(Instance i)
```

:   Gets the index of the branch that an instance applies to. Returns -1 if no branches
    apply.

    :   **Parameters:**: `i` - the instance **Returns:**: the branch index

---


### instancesDownBranch

```
public abstract ReferenceInstances instancesDownBranch(int branch,
                                                       Instances sourceInstances)
```

:   Gets the subset of instances that apply to a particluar branch of the split. If the
    branch index is -1, the subset will consist of those instances that don't apply to
    any branch.

    :   **Parameters:**: `branch` - the index of the branch: `sourceInstances` - the instances from which to find the subset **Returns:**: the set of instances that apply

---


### attributeString

```
public abstract java.lang.String attributeString(Instances dataset)
```

:   Gets the string describing the attributes the split depends on.
    i.e. the left hand side of the description of the split.

    :   **Parameters:**: `dataset` - the dataset that the split is based on **Returns:**: a string describing the attributes

---


### comparisonString

```
public abstract java.lang.String comparisonString(int branchNum,
                                                  Instances dataset)
```

:   Gets the string describing the comparision the split depends on for a particular
    branch. i.e. the right hand side of the description of the split.

    :   **Parameters:**: `branchNum` - the branch of the split: `dataset` - the dataset that the split is based on **Returns:**: a string describing the comparison

---


### equalTo

```
public abstract boolean equalTo(Splitter compare)
```

:   Tests whether two splitters are equivalent.

    :   **Parameters:**: `compare` - the splitter to compare with **Returns:**: whether or not they match

---


### setChildForBranch

```
public abstract void setChildForBranch(int branchNum,
                                       PredictionNode childPredictor)
```

:   Sets the child for a branch of the split.

    :   **Parameters:**: `branchNum` - the branch to set the child for: `childPredictor` - the new child

---


### getChildForBranch

```
public abstract PredictionNode getChildForBranch(int branchNum)
```

:   Gets the child for a branch of the split.

    :   **Parameters:**: `branchNum` - the branch to get the child for **Returns:**: the child

---


### clone

```
public abstract java.lang.Object clone()
```

:   Clones this node. Performs a deep copy, recursing through the tree.

    :   **Overrides:**: `clone` in class `java.lang.Object`
    :   **Returns:**: a clone


---


|  |  |  |
| --- | --- | --- |
| **Overview** \| **Package** \| **Class** \| **Tree** \| **Deprecated** \| **Index** \| **Help** \| **Weka's home** | |  |
| **PREV CLASS**   **NEXT CLASS** | **FRAMES**    **NO FRAMES**     **All Classes** |  |
| SUMMARY: NESTED \| FIELD \| CONSTR \| METHOD | DETAIL: FIELD \| CONSTR \| METHOD |  |


---
